# Impact of clinical factors and allograft leukocyte content on post-transplant lymphopenia, monocytopenia, and survival in patients undergoing allogeneic peripheral blood haematopoietic cell transplant

**DOI:** 10.1186/2052-1839-14-14

**Published:** 2014-09-01

**Authors:** Mary D Thoma, Jennifer Glejf, Eapen Jacob, Tanya J Huneke, Lori J DeCook, Nicci D Johnson, Mrinal M Patnaik, Mark R Litzow, William J Hogan, Laura F Newell, Rekha Chandran, Luis F Porrata, Shernan G Holtan

**Affiliations:** Department of Medicine, Division of Hematology, Mayo Clinic Graduate School of Medicine, Rochester, MN USA; Department of Pathology and Laboratory Medicine, Mayo Clinic Graduate School of Medicine, Rochester, MN USA; Department of Medicine, Division of Hematology, Mayo Clinic Graduate School of Medicine, Phoenix, AZ USA; Medical College of Wisconsin, Milwaukee, WI USA; Center for Hematologic Malignancies, Knight Cancer Institute, Oregon Health & Science University, Portland, OR USA; Blood and Marrow Transplant Program, University of Minnesota, 420 Delaware Street SE, Minneapolis, MN 55455 USA

**Keywords:** Allogeneic haematopoietic cell transplantation, Lymphopenia, Monocytopenia, GVHD, CMV viraemia

## Abstract

**Background:**

We have previously shown that lymphopenia and monocytopenia at 2–3 months post-allogeneic haematopoietic cell transplant (HCT) is associated with poor survival in recipients of both myeloablative and reduced intensity conditioning regimens. It is not known whether the graft leukocyte content has a role in early lymphocyte and monocyte recovery following allogeneic T-cell replete peripheral blood HCT.

**Methods:**

Haematologic recovery data, including absolute lymphocyte and monocyte counts (ALC and AMC, respectively) at day +15, +30, +60, and +100, and outcomes data were pooled from two prior independent cohorts, and parameters were correlated with leukocyte graft content in those individuals receiving peripheral blood progenitor cell grafts. 216 consecutive patients from 2001–2010 were included in the analysis.

**Results:**

Neither infused allograft lymphocyte, monocyte, granulocyte, nor CD34+ cell number per kilogram recipient body weight correlated with haematologic recovery parameters or overall survival in this cohort. Prognostic factors for overall survival based on multivariate analysis were as expected from the results of the previous independent cohorts and included severity of chronic GVHD (p < 0.001), development of post-transplant relapse (p = 0.001), day +60 AMC > 0.3 x 10^9^ cells/L (p = 0.0015), and day +100 ALC > 0.3 x 10^9^ cells/L (p < 0.001). Low monocyte and lymphocyte counts at the day +60 and day +100 time points were significantly associated with acute GVHD and/or CMV viraemia.

**Conclusions:**

This study suggests that graft cell count does not influence post-transplant monocyte and lymphocyte recovery following T-cell replete allogeneic peripheral blood HCT. Post-transplant complications such as acute GVHD and/or CMV viraemia negatively influenced monocyte and lymphocyte recovery, and hence the survival. Further studies aimed at understanding the mechanisms behind sustained lymphopenia and monocytopenia post-transplant are needed.

## Background

The development of cytopenias after initial donor haematopoietic cell engraftment is a risk factor for poor transplant outcomes [1]. We have previously identified that significant lymphopenia and monocytopenia, defined as an absolute lymphocyte or monocyte count (ALC and AMC, respectively) of < 0.3 × 10^9^ cells/L at day +100 post-transplant, are independent risk factors for inferior survival [2, 3]. The objective of this study was to determine whether the leukocyte content of the T-cell replete peripheral blood haematopoietic cell allograft is responsible at least in part for post-transplant ALC and AMC recovery in the first 100 days post-HCT, or whether the secondary cytopenias predominantly reflect the detrimental impact of transplant complications on graft function and immune reconstitution. Allograft monocytes have previously been shown to suppress T-cell proliferation in a dose-dependent fashion, with an associated decrease in acute GVHD risk in retrospective series [4, 5]. A previous prospective study of allograft leukocyte content did not identify an association between pot-transplant survival and lymphoid and myeloid reconstitution [6], although monocytes were not analyzed in that study. Clarification of the role of infused graft monocytes in controlling acute graft versus host disease (GVHD) could lead to modification of apheresis strategies or other opportunities for allograft engineering to reduce transplant complications.

## Methods

### Patient characteristics

Patients included in this analysis were pooled from our prior two reports, a myeloablative (MA) cohort of 135 patients, and a reduced intensity (RIC) cohort of 118 patients [2, 3]. Patients receiving bone marrow grafts were eliminated from the analysis (24 from the MA and 8 from the RIC cohorts). Of the 221 remaining patients, 5 did not have complete data on graft leukocyte subsets, leaving 216 consecutive patients available for analysis in this study. All patients underwent allogeneic HCT from 2001-2010 at Mayo Clinic in Rochester, MN. All patients gave written consent to use their medical records for research. No patients were lost to follow-up. Approval for the retrospective review of these records was obtained from the Mayo Clinic Institutional Review Board and was in accordance with US federal regulations and the Declaration of Helsinki.

### Determination of allograft leukocyte subset content

Lymphocyte, granulocyte, and monocyte content was obtained prior to 11/13/2009 by manual differentials using standard clinical criteria. After that time, flow cytomtery using a BD FACSCalibur (Becton Dickinson Biosciences, San Jose, California) was used to determine product content using forward and side scatter characteristics of the lymphocyte, granulocyte and monocytes. This method was validated in the laboratory and proven to be consistent with prior results with manual differentials. CD34 positive cell content was determined by flow cytometry using the Stem-Kit (Beckman Coulter, Brea, California) in combination with BD TruCount beads in a lab developed test. All data were obtained from results generated during routine clinical care.

### End points

The primary end point of the study was to determine the association of allograft leukocyte content on overall survival, post-transplant complications, and peripheral blood ALC and AMC at day +15, +30, +60, and +100 post- HCT. Enumeration of leukocyte subsets, including CD34+ cells, granulocytes, monocytes, and lymphocytes was performed on fresh allografts after apheresis and prior to transplant per institutional standard operating procedures. The ALC and AMC were obtained by review of the medical records of the complete blood cell count (CBC) [7] at day +15, +30, +60, and +100 post-HCT.

### Prognostic factors and survival

The prognostic factors evaluated in the study included: graft leukocyte subset content, age at transplant, disease status at transplant, related versus unrelated donor stem cell source, degree of HLA-matching, treatment of confirmed, quantifiable CMV viraemia by PCR with ganciclovir or valganciclovir prior to day +100, presence/grade of acute GVHD based upon the Glucksberg-Seattle grading system [8], presence/severity of chronic GVHD based upon NIH consensus criteria [9] and ALC and AMC recovery post-HCT. Overall survival (OS) was defined from the time of transplant to last follow-up or death due to any cause.

### Statistical analysis

OS estimates were determined using the approach of Kaplan and Meier [10]. Differences between survival curves were tested for statistical significance using the 2-tailed log-rank test. The Cox proportional hazard model was used for univariate analysis of continuous variable as well as multivariate analysis. Graft leukocyte subsets were analyzed as continuous variables. Dividing the graft subsets in half, in tertiles, or in quartiles, did not change the results of the analysis (not shown). Associations between categorical variables were determined by Fisher’s exact test. Correlations between continuous variables were determined by Spearman’s rho. Because of multiple comparisons with the correlation analysis, a Bonferroni correction was applied. All p-values represented were two-sided and statistical significance was declared at p< 0.05.

## Results and discussion

### Patient characteristics

Patient characteristics are described in Table 1. The median age of the cohort was 51 years with a slight female predominance (54%). The underlying disease was AML in 53%. All patients received consistent supportive care including calcineurin inhibitor-based graft-versus-host disease prophylaxis. The majority of the stem cell grafts were derived from fully-matched related donors (86%). Over half of patients underwent allogeneic HCT in first remission (58%). Median overall survival for the entire cohort was 26.6 months. 109 patients died at the time of analysis, and the median follow up for surviving patients was 50.1 months. 68 patients (31%) in this cohort died from non-relapse complications, and 41 patients (19%) died from relapse.

**Table 1:** **Patient characteristics and univariate survival analysis**

Characteristic		Patients (N = 216)	%	Univariate OS p
Year of transplant, median (range)		2007 (2000–2010)		0.03
Age, median (range)		51 (19–71)		0.44
Sex	Male	99	46%	0.42
	Female	117	54%	
Conditioning	FM (RIC)	109	50%	0.55
	MA (Cy/TBI or Bu/Cy)	107	50%	
Disease	AML	115	53%	0.05
	MDS	32	15%	
	NHL	11	5%	
	MM	9	4%	
	ALL	41	19%	
	CML	4	2%	
	HL	2	1%	
	CLL	1	0%	
	MPN	1	0%	
Donor type	Related	186	86%	0.47
	Unrelated	30	14%	
Match Degree	Complete match	204	94%	0.92
	1 allele or antigen mismatch	10	5%	
	2 allele or antigen mismatch	2	1%	
Disease status at transplant	Complete remission	126	58%	0.003
	Not in remission	90	42%	
CD34+ cells x 10(6)/kg infused, median (range)		6.0 (1.85 - 17.06)		0.74
Monocytes x 10(6)/kg infused, median (range)		250 (25.7 - 1,139)		0.27
Lymphocytes x 10(6) infused, median (range)		419 (3.04 - 1,530)		0.92
Granulocytes x 10(6) infused, median (range)		166 (5.3 - 1,038)		0.4
Days to ANC 500, median (range)		15 (3–101)		0.83
Days to platelet 50,000 median (range)		19 (0–393)		0.67
Maximal grade of acute GVHD	0	99	46%	<0.001
	1	35	16%	
	2	49	23%	
	3	24	11%	
	4	9	4%	
Severity of chronic GVHD	None	89	41%	<0.001
	Mild	32	15%	
	Moderate	51	24%	
	Severe	44	20%	
CMV reactivation	Yes	72	33%	0.15
	No	144	67%	
Relapse	Yes	47	22%	0.002
	No	169	78%	
ALC day +15 > 0.3 x 10(9)/L		97	45%	0.017
AMC day +15 > 0.3 x 10(9)/L		142	66%	0.94
ALC day +30 > 0.3 x 10(9)/L		183	85%	<0.001
AMC day +30 > 0.3 x 10(9)/L		193	89%	0.001
ALC day +60 > 0.3 x 10(9)/L		172	80%	0.0004
AMC day +60 > 0.3 x 10(9)/L		138	64%	<0.001
ALC day +100 > 0.3 x 10(9)/L		158	73%	<0.001
AMC day +100 > 0.3 x 10(9)/L		139	64%	<0.001

### Allograft leukocyte content, ALC and AMC recovery, and survival: Univariate analysis

No graft leukocyte subset was associated with OS as a continuous variable (Table 1), or divided above/below the median, or in quartiles (not shown). As expected from our prior reports, ALC and AMC >0.3 × 10^9^/L was associated with improved survival, especially at the day +30, 60, and day +100 time points. Graft leukocyte subsets were not associated with the development of the clinical outcomes of acute GVHD, chronic GVHD, CMV reactivation, or relapse (Table 2). Graft leukocyte subsets did not correlate with post-transplant ALC or AMC at any of the time points measured (Table 3).

**Table 2:** **Association of graft leukocytes with clinical outcomes**

Category	Cells/kg	p
Acute GVHD	Graft monocytes	0.35
	Graft lymphocytes	0.08
	Graft granulocytes	0.56
	Graft CD34+	0.62
Chronic GVHD	Graft monocytes	0.18
	Graft lymphocytes	0.65
	Graft granulocytes	0.4
	Graft CD34+	0.24
CMV reactivation	Graft monocytes	0.1
	Graft lymphocytes	0.14
	Graft granulocytes	0.32
	Graft CD34+	0.07
Relapse	Graft monocytes	0.23
	Graft lymphocytes	0.51
	Graft granulocytes	0.28
	Graft CD34+	0.1

**Table 3:** **Associated variables**

Variable	By variable	Spearman’s rho	p
D15AMC	D15ALC	0.65	<.0001
Graft Mono/kg	Graft Lymph/kg	0.61	<.0001
Graft Gran/kg	Graft Mono/kg	0.53	<.0001
D100ALC	D60ALC	0.56	<.0001
D30AMC	D30ALC	0.50	<.0001
Graft Gran/kg	Graft Lymph/kg	0.48	<.0001
D100AMC	D100ALC	0.50	<.0001
D30ALC	D15ALC	0.40	<.0001
D60ALC	D30ALC	0.38	<.0001
D100AMC	D60AMC	0.38	<.0001
D60AMC	D60ALC	0.36	<.0001
D100ALC	D30ALC	0.30	0.014
D60ALC	D15ALC	0.28	0.02
D30AMC	D15ALC	0.27	0.02
D100ALC	D15ALC	0.28	0.03
D100AMC	D15AMC	0.28	0.05

Only statistically significant relationships are shown

### Multivariate analysis

Multivariate analysis (Table 4) including the parameters that demonstrated a significant difference in overall survival in the univariate analysis revealed the following independent prognostic factors: severity of chronic GVHD, development of post-HCT relapse, day +60 AMC, and day +100 ALC. Day +60 monocytopenia occurred in 57 patients and was associated with day +100 lymphopenia (p = 0.05), which occurred in 18 patients.

**Table 4:** **Multivariate analysis**

	RR	95% CI	P
cGVHD severity			<0.001
mild vs. none	0.3	0.12 - 0.64	
mild vs. moderate	0.88	0.32 - 2.3	
mild vs. severe	0.26	0.1 - 0.68	
Relapse	2.7	1.64 - 4.35	0.0001
D60 AMC > 0.3 x 10(9)/L	0.44	0.26 - 0.73	0.0015
D100 ALC > 0.3 x 10(9)/L	0.22	0.11 - 0.46	<0.001

### Association of monocytopenia, lymphopenia, and GVHD

Acute GVHD was associated with day +60 monocytopenia (median 0.2 vs. 0.3 × 10^9^/L, p = 0.01) and less so with day +100 lymphopenia (median 0.5 vs. 0.6 × 10^9^/L, p = 0.05).

### Association of monocytopenia, lymphopenia, and CMV

CMV reactivation was strongly associated with day +60 monocytopenia but no other longitudinal haematopoietic parameter in this study (median 0.1 vs. 0.4 × 10^9^/L for those who did vs. did not receive treatment for CMV reactivation prior to day +100, p < 0.001).

### Association of monocytopenia, lymphopenia, and relapse

Day +100 ALC was lower in patients who experienced subsequent relapse of their underlying malignancy (0.3 vs. 0.6 × 10^9^/L, p = 0.03). No other associations between cytopenias and transplant complications were identified at the other time points studied.

### Association of monocytopenia, lymphopenia, overlapping transplant complications, and long term survival

**Figure 1:**
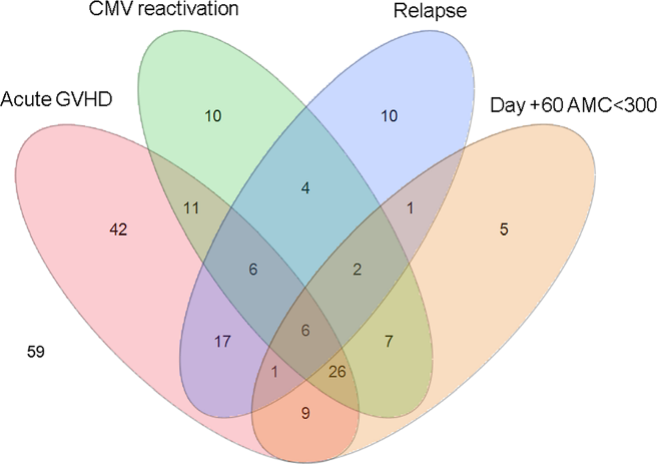
**Venn diagram of patients experiencing post-transplant complications in association with day +60 monocytopenia.**

**Figure 2:**
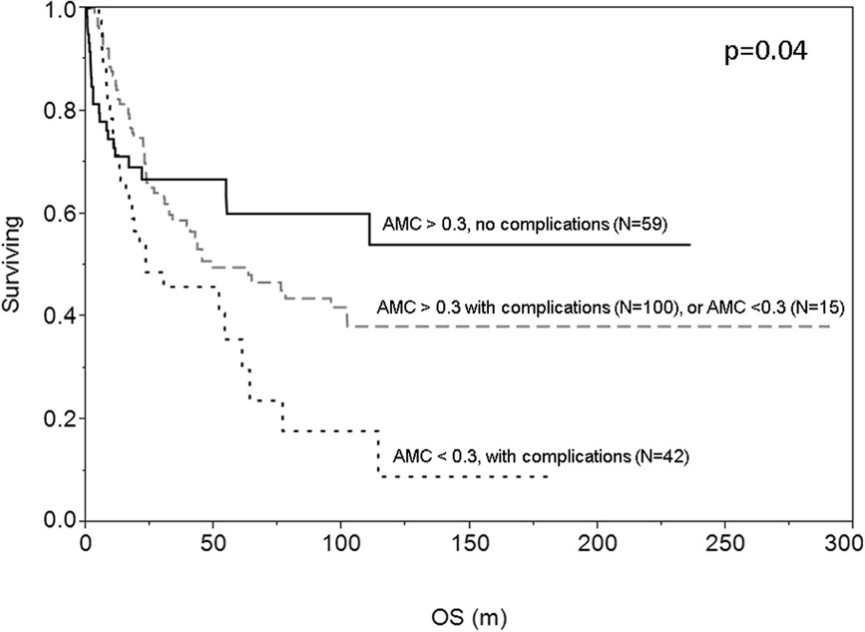
**Kaplan Meier estimate of patients experiencing post-transplant complications in association with day +60 monocytopenia.**

**Figure 3:**
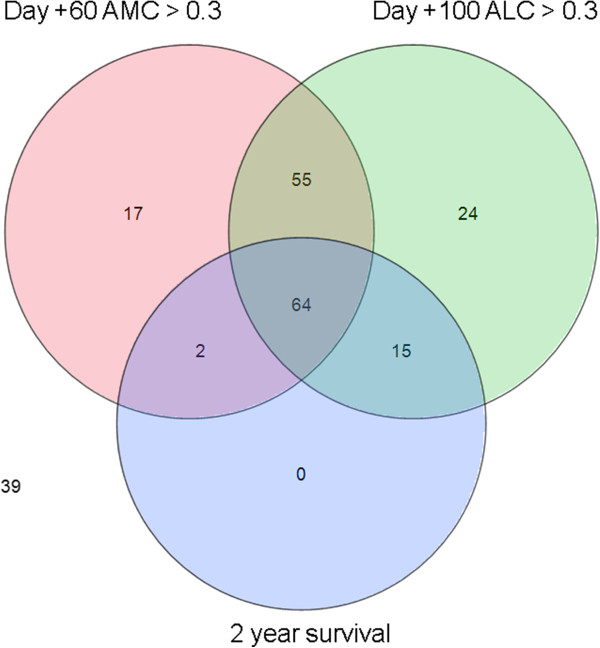
**Two year survival in association with day +60 AMC and day +100 ALC thresholds.**

Recognizing that patients can have multiple complications at once, we constructed a Venn diagram that depicts overlapping of post-transplant complications (acute GVHD, CMV reactivation, and relapse) in patients with the most prevalent prognostic laboratory value in this series, day +60 monocytopenia (Figure 1). Over 90% of the 57 patients with day +60 monocytopenia had concurrent acute GVHD and/or CMV reactivation (Figure 1). Importantly, day +60 monocytopenia in the setting of acute GVHD and CMV reactivation negatively impacted survival (Figure 2). In an effort to understand additional risk factors for these secondary cytopenias, increasing age showed a trend toward failure to achieve the 0.3 × 10^9^/L AMC threshold at day +60 (p = 0.07), but no other host or graft factors could be identified at this or other time points for ALC or AMC. All 2 year survivors (81 patients, 38%) met the predetermined either day +60 AMC or day +100 ALC threshold of >0.3 × 10^9^/L, and majority of them met both (Figure 3). Of the 55 patients who met both ALC and AMC thresholds but did not survive 2 years, 41 of them (75%) died from relapsed disease. 39 patients who did not meet the ALC or AMC thresholds did not survive 2 years.

### Is there an optimal cellular composition of allografts?

In this report, we demonstrate that post-allogeneic HCT lymphopenia and monocytopenia in the intermediate recovery phase post-HCT are related to early complications rather than allograft leukocyte content. This is in contrast to the autologous transplantation setting, where the autograft lymphocyte and monocyte content directly correlates with post-transplant lymphocyte and monocyte recovery and survival [11–14]. In our cohort, we identified age as a possible contributor to post-transplant secondary cytopenias, as have others [1]. Similarly, a recent analysis of lymphocyte populations in patients enrolled in Blood and Marrow Transplant Clinical Trials Network trial 0302 also identified that patients responding to therapy for acute GVHD, regardless of the treatment arm, had a less substantial decline in peripheral blood CD45+ leukocytes compared to those who were not responding to treatment [15]. However, other studies have identified a correlation with various components of allografts and post-HCT hematopoiesis and outcomes [6, 16–19]. It is possible that more refined subsets other than simple granulocyte, monocyte, and lymphocyte content of the graft relates to outcomes. Lonial et al recently identified that granulocyte-macrophage colony stimulating factor given concomitantly with G-CSF mobilized fewer plasmacytoid dendritic cells and T cells from HLA-matched sibling donors, leading to more rapid immunologic recovery but with no overall impact on survival [20].

### What are the mechanisms behind post-HCT cytopenias?

Medications, especially steroids and ganciclovir, are well known to cause cytopenias, predominantly lymphopenia with the former and neutropenia with the latter, although such side effects are not universal among HCT recipients. Efforts to better understand other mechanisms of post-transplant cytopenias are ongoing, including the potential role of pro-inflammatory cytokines such as tumor-necrosis factor-alpha (TNF-α) and interferon-gamma (IFN-γ) on the suppression of hematopoiesis occurring after HCT. As previously described, chemotherapy and radiation conditioning regimens administered prior to HCT lead to damage of host tissues, production of pro-inflammatory cytokines [i.e. TNF-α, interleukin 1 and 6 (IL-1, IL-6)] and other inflammatory stimuli (i.e. lipopolysaccharide, LPS), which then promotes the activation of antigen-presenting cells (APCs) involved in the pathogenesis of acute GHVD [21]. Pro-inflammatory cytokines, and specifically TNF-α and IFN-γ, have also been implicated in the pathogenesis of primary bone marrow failure states and haematopoietic stem cell suppression [22–26] and the inflammatory state that develops in the setting of post-HCT complications such as GVHD or infections. Why some patients are more susceptible to secondary cytopenias and marrow suppression, as well as other post-HCT complications, is unclear but may relate to patient-specific and disease-specific differences such as specific single-nucleotide polymorphisms as well as non-HLA genetic variation within the major histocompatibility complex [27–31] or other mechanisms. The role of these patient and disease variables, as well as the contribution of cytokine fluctuations [32], on post-HCT cytopenias, immunologic reconstitution, as well as post-HCT outcomes, is an area of active investigation and remains to be fully elucidated.

### Limitations

Our study has several limitations. This retrospective cohort was transplanted at a single institution, where the majority of transplants were from HLA-matched sibling donors. These results may not be applicable to the minimal intensity setting, nor to cohorts where the majority of grafts are derived from unrelated or alternate donors, although early lymphocyte recovery has previously shown to be prognostic after T-cell depleted grafts, [33] umbilical cord blood grafts [34], and unmanipulated haploidentical grafts [35]. In the latter study, a lymphocyte recovery threshold of >0.3 × 10^9^/L at day +30 was also shown to significantly impact OS, PFS, relapse and TRM and was significantly correlated with CD3+ T cells in the allograft [35]. Additionally, detailed flow cytometric phenotyping of cellular subsets was also not performed (e.g., graft regulatory T cells [36]), and all data was generated from that encountered during routine clinical care. Therefore to confirm the findings of this study, a larger retrospective study using CIBMTR database of over 30,000 HCT procedures is being performed. While confirmatory studies are underway, this study suggests that both monocytopenia and lymphopenia at 2-3 months post-HCT are emerging predictors of poor survival. Development of these secondary cytopenias appears to be related to the development of acute GVHD and CMV viraemia and possibly recipient age, as opposed to graft leukocyte content or other established clinical factors.

Although allogeneic transplantation offers the only curative option for many advanced and high-risk haematologic malignancies, the costs associated with the procedure have come under increased scrutiny ($96,000 -$204,000 in the first year in 2012) [37]. The single biggest driver of expenditure is the length of inpatient hospital stay [37]. The role of cytopenias is particularly important given its associations with acute GVHD and infections, both of which frequently necessitate hospital admissions [38]. Patients with secondary cytopenias may be those requiring increased resource utilization. Therefore, this group of patients may be appropriate for early, novel interventions to facilitate haematologic and immune recovery. If our findings are confirmed in larger studies, it would provide a rationale for conducting clinical trials to proactively enhance lymphohaematopoietic recovery following allogeneic HCT.

## Conclusions

In our cohort of 216 recipients of peripheral blood progenitor cell allografts, we did not identify a clear role of graft monocyte or other cellular component as being predictive of GVHD, relapse, or survival. Post-transplant lymphopenia and monocytopenia were predominantly associated with acute GVHD and preemptive therapy for CMV viraemia. In patients experiencing these complications but with adequate lymphocyte and monocyte counts at the day +60 and day +100 time points, long term survival did not appear to be negatively impacted. Further studies aimed at understanding which patients are most vulnerable to developing secondary cytopenias, and how to improve immune recovery in the face of these complications, are needed.

## Abbreviations

ALC: Absolute lymphocyte count
ALL: Acute lymphoblastic leukemia
AMC: Absolute monocyte count
AML: Acute myeloid leukemia
ANC: Absolute neutrophil count
CLL: Chronic lymphocytic leukemia
CI: Confidence interval
CML: Chronic myeloid leukemia
CMV: Cytomegalovirus
GVHD: Graft versus host disease
FM: Fludarabine and melphalan
HCT: Haematopoietic cell transplant
HL: Hodgkin lymphoma
HLA: Human leukocyte antigen
KG: Kilogram
MA: Myeloablative
MM: Multiple myeloma
MDS: Myelodysplastic syndrome
MPN: Myeloproliferative neoplasm
NHL: Non-Hodgkin lymphoma
OS: Overall survival
RR: Relative risk.
